# Editorial: Deciphering the Microbiome-Immunity-Cancer Axis

**DOI:** 10.3389/fimmu.2022.897811

**Published:** 2022-04-14

**Authors:** Xin Chen, Chengcheng Jin, Andrea Facciabene, Xiaohuan Guo, Yanbo Zhang

**Affiliations:** ^1^ School of Medicine, Tsinghua University, Beijing, China; ^2^ Department of Cancer Biology, Perelman School of Medicine, University of Pennsylvania, Philadelphia, PA, United States; ^3^ Department of Radiation Oncology and the Ovarian Cancer Research Center, Perelman School of Medicine, University of Pennsylvania, Philadelphia, PA, United States; ^4^ Institute for Immunology, Tsinghua University, Beijing, China; ^5^ Department of Immunology, Harvard Medical School, Boston, MA, United States; ^6^ Evergrande Center for Immunologic Diseases, Harvard Medical School and Brigham and Women’s Hospital, Boston, MA, United States

**Keywords:** microbiome, immunity, cancer, CRC, lung cancer, pancreatic cancer, metabolites

Trillions of microbes inhabit human body surfaces and cavities and interact with the host constantly. Recently, it came to light that the microbiota is a critical regulator of tumorigenesis and impacts outcomes of various cancer therapies. Pathogenic bacteria can contribute to cancer progression *via* inducing a variety of tumor-promoting inflammatory responses while beneficial commensals prevent inflammation and stimulate the host anti-cancer immunity. Hence, a delicate balance between the microbiomes and the immune system plays critical roles in the development, prevention and therapy of cancer.

In this Research Topic, 8 articles cover different aspects of the microbiome-immunity-cancer interplay in different indications including colorectal cancer (Fidelle et al. and Hanus et al.), lung cancer (Dong et al. and Feng et al.), pancreatic cancer (Alkharaan et al. and Huang et al.), as well as new discoveries in pre-clinical models (Han et al. and Huang et al.). In addition, Jain et al. provide a comprehensive overview of the decades of the discoveries in the cancer-microbiome-immune axis.

Cancer ranks as a leading cause of death worldwide. Among all cancer types, colorectal cancer (CRC) is the third most common malignancy and second leading cause of cancer mortality (1). The colon harbors the majority of bacteria in the body and contains the largest pool of immune cells from innate and adaptive arms. Rather than being composed of homogeneous malignant cells, the CRC tumor microenvironment is a highly heterogeneous contexture. As comprehensively described in the review by Fidelle et al., CRC arises from progressive accumulations of genetic and epigenetic alterations and is also dictated by intestinal immune system and microbiome. Dysbiosis is associated or causally linked with colon carcinogenesis. Bacteria such as *Fusobacterium nucleatum*, *Escherichia coli* and *Enterotoxigenic Bacteroides fragilis (ETBF)* are closely associated with and modulate tumor progression through multiple mechanisms. Of note, as discussed by Fidelle et al., Hanus et al. and Jain et al., microbial metabolites, such as short-chain fatty acids (SCFAs) and polyamines metabolites may herein play critical roles. Furthermore, as suggested by Fidelle et al. CRC therapies and gut microbiota interplay with each other. Distinct chemotherapeutic regimens, including Oxaliplatin, capecitabine, and Gemcitabine, influence the composition of the gut microbiota, and in turn, the microbes could influence the effect of the therapy, by modulating the immune response, metabolizing the drugs, or interfering with tumor signaling pathways, and impact clinical outcomes. In addition, accumulating evidence suggested pivotal roles of the gut microbiome in the efficacy of immune checkpoint inhibitors (ICIs), as discussed by Fidelle et al. and Jain et al. Intriguingly, as proposed by Fidelle et al., ileal epithelial cells and its microbial ecosystem, may play a central role in mobilizing an efficient immune response against self-antigens from colon cancers. Cytotoxic regimen inducing immunogenic cell death (ICD) of tumor and/or crypt stem cells, with achievement of humoral and cellular immune responses and a proper ileal microbiome composition balancing immunogenic and tolerogenic commensals may serve as the solution to the paradox of colon cancers. Moreover, potential clinical significance of new players, such as T follicular helper cells (Tfh), B cells, and antibodies to self-antigens are highlighted.

Albeit the gastrointestinal tract serves as the primary habitat of human commensal bacteria, the lung, harboring one of the largest interfaces between the human body and external environment, is another unique site for host-microbiome interaction. Lung cancer is the second most frequently diagnosed cancer and the leading cause of cancer death worldwide (1). The review by Dong et al. highlights the landscape of lung microbiome and their association with lung cancer. As discussed by the authors, the lung possesses unique bacteria compositions which are different from those of the gut or skin. Lung cancer is frequently associated with local dysbiosis. Specific bacterial taxa may impact the tumorigenesis by direct regulating ontogenetic pathways (e.g., *via* microbial metabolites), or by modulating immune equilibrium between suppression and activation. Besides the local cancer-immune-microbiome axis in lung, the authors also bring forward the gut-lung axis and propose that distal intestinal microbiota may indirectly regulate lung cancer development and therapeutic outcomes of chemotherapy or immunotherapy through modulating systemic immune response. Moreover, as our understanding of how the microbiome co-evolve with tumors improves, the lung and gut microbiome may serve as robust prognostic biomarkers and provide new therapeutic strategies for lung cancer treatment. From another perspective, Feng et al., reported the prognostic significance of gene signature of tertiary lymphoid structures (TLS) in patients with lung adenocarcinoma (LUAD) in an original research article. The study provided evidence that high TLS signature in LUAD is associated with favorable immune responses and better prognosis of the patients, suggesting TLS as an independent prognostic marker for LUAD.

Besides CRC and lung cancer, pancreatic cancer is another highly lethal cancer type in which the influence of microbiome on tumor immune infiltration and cancer outcomes has been reported (2). Oral commensals, including *Fusobacterium* species in pancreas are described to be associated with a worse prognosis of pancreatic cancer (3) and are enriched in intraductal papillary mucinous neoplasm with high-grade dysplasia (IPMN HGD) (4). However, the association of circulating and salivary antibodies against commensal bacteria with the pancreatic cancer development was not clear. In this Research Topic, the study by Alkharaan et al. brings interesting data to support a positive correlation of plasma IgG and salivary IgA reactivity to *Fusobacterium nucleatum* with IPMN HGD, suggesting a predictive role of humoral response against pancreas-associated oral bacteria in IPMN severity and risk of pancreatic cancer. From another angle of view to understand the influence of microbiota on pancreas cancer, Huang et al. analyzed the intratumor microbiome and identified a positive association between *Megasphaera* and the survival time of Chinese patients with pancreatic ductal adenocarcinomas (PDAC), suggesting *Megasphaera* as a prognostic and therapeutic target for the treatment of PDAC.

In a pre-clinical study, Han et al. reported the effect of antibiotics and microbial metabolites on the anti-tumor activity of γδT cells. As shown by the authors, antibiotics treatment benefits the γδT cell therapy in a HepG2 xenograft mouse model, and 3-indopropionic acid serves as a potential intermediate molecule to connect the altered gut microbiota and γδT cells.

Collectively, the original research papers and reviews presented in this Research Topic provide a comprehensive overview of the triangle of microbiome-immunity-cancer interactions and highlight the clinical significance of microbes in a wide spectrum of cancer prognosis and treatments. The insights outlined here shed light on future directions in manipulating microbiome and immunity towards a cure of cancer from bench side to clinic.

## Author Contributions

XC drafted the manuscript. All authors were involved in the critical revision of the manuscript and approved the submitted version.

## Conflict of Interest

The authors declare that the research was conducted in the absence of any commercial or financial relationships that could be construed as a potential conflict of interest.

## Publisher’s Note

All claims expressed in this article are solely those of the authors and do not necessarily represent those of their affiliated organizations, or those of the publisher, the editors and the reviewers. Any product that may be evaluated in this article, or claim that may be made by its manufacturer, is not guaranteed or endorsed by the publisher.
